# Profile and Motivation of Patients Consulting in Emergency Departments While not Requiring Such a Level of Care

**DOI:** 10.3390/ijerph16224431

**Published:** 2019-11-12

**Authors:** Daniel Aiham Ghazali, Arnaud Richard, Arnaud Chaudet, Christophe Choquet, Maximilien Guericolas, Enrique Casalino

**Affiliations:** 1 Emergency Department, University Hospital of Bichat, 75018 Paris, France; mrarnorichard@outlook.fr (A.R.); christophe.choquet@aphp.fr (C.C.); maximilien.guericolas@aphp.fr (M.G.); enrique.casalino@aphp.fr (E.C.); 2 Study Group for Efficiency and Quality of Emergency Departments and Non-scheduled Activities Departments, 75018 Paris, France; 3 Simulation Center, University of Paris Diderot, 75018 Paris, France; 4 INSERM UMR 1137, IAME, University of Paris Diderot, 75018 Paris, France; 5 Emergency Department, University Hospital of Poitiers, 86000 Poitiers, France; arnaud.chaudet@outlook.com

**Keywords:** emergency department, non-urgent consult, profile, motivation, overcrowding

## Abstract

Consultations that do not require an emergency department (ED) level of care have increased. We explored attitudes of non-urgent patients in two academic hospitals in France with a similar fast track organization. One of them is a Parisian hospital with 90,000 patients/year who are admitted to the ED, while the other admits 40,000 patients/year in a smaller city. During one month in 2018, the triage nurse handed out a survey to patients coming for non-urgent consultations. It was given back to the fast track physician at the end of the visit; 598 patients agreed to answer. They were mostly young males with adequate social coverage, consulting for osteo-articular pathologies, without any significant difference between the two sites (*p* = 0.32). They were equally satisfied with the care they received (*p* = 0.38). Satisfaction was inversely correlated to waiting time (*p* < 0.0001). Convenience, accessibility of emergency facilities, and geographic proximity were motivation factors. These results suggest that primary care providers who can access testing facilities in accordance with patient needs might be a solution to help reduce overcrowding in EDs.

## 1. Introduction

### Definition of a Consultation not Requiring a Medical Emergency Department Level of Care

Many patients who consult at an emergency department (ED) do not require immediate diagnosis or treatment, and therefore do not require care in an ED. Data on these patients throughout literature are very heterogeneous due to the absence of a consensus on how to define these particular patients. The literature review by Durand et al. highlighted the confusion between consultations considered non-urgent and those considered inappropriate [1]. The concept of a non-urgent consultation is tied to the severity of the pathology, speed of evolution, or need for hospitalization or not. The concept of an inappropriate consult is tied to the social and psychological context on top of the medical issue, and must integrate factors such as visiting hours and availability of healthcare services in the vicinity of the ED [1]. It, therefore, seems more appropriate to use the terminology of consultations not requiring an ED level of care that are manageable in a primary care setting.

Non-urgent visits to EDs are a controversial issue as they have been negatively associated with crowding and cost [1]. A steady increase in health-establishment-based ED visits has been observed in France. Since 2002, EDs have reported a 40% to 50% increase in visits [2]. This increase cannot be attributed solely to demographic growth or to an aging population. Some patients, particularly homeless people, migrants, refugees, and asylum seekers, have difficulty choosing between different means of access to health services. Despite the crisis in Europe in 2008, immigration has increased by 2% per year [3]. This phenomenon has been accentuated in Europe, including France, due to wars in the Middle East and Africa. Over recent years, there has been a 35% increase of asylum seekers in Europe [4]. There exist differences in healthcare service utilization between immigrant and native populations [5]. In France, for both cultural and administrative reasons, social analyses by ethnic origin are not routinely carried out [6]. However, immigrants present a lower demand for GP and specialist care [7]. In Europe, they seem to more frequently prefer emergency healthcare services than native born patients [4,5]. Unfortunately, accurate registration regarding healthcare services provided to undocumented migrants is largely lacking. Nevertheless, a recent systematic review showed that they tended to underutilize several types of healthcare services [8].

The volume of consultations not requiring an ED level of care has increased in other developed countries [9]. These “inappropriate” consultations represent 10% to 90% of all visits, with a median of 32% [1]. Non-urgent ED visits are a major problem in several countries, including Kuwait (61%), Cuba (57.9%), and Hong Kong (57.0%). In other countries they are moderate, such as Germany (49.9%), Great Britain (40.9%), Sweden (38.3%), France (31.7%), Portugal (31.3%), and Turkey (31.2%). In still other countries they are less prevalent, such as the United States (12.5%), Italy (19.6%), Brazil (24.2%), Canada (25%), and Spain (29.6%). This spread is due to the absence of a universal definition for “non-urgent consultations”. These visits are all the more “inappropriate” because the majority of them occur during the business hours of most primary care providers (PCP) [10], for the most part at the sole initiative of the patient [1,2,11,12,13]. Personal convenience and ease of emergency care use were listed as motivations for consulting ED in several countries, such as Japan [14], the United States [15], and Spain [16]. This influx of non-severe patients does not benefit from the holistic management that a general practice provides and it increases the already growing number of ED consultations caused by an aging population and a rise in chronic illnesses [17].

This growing phenomenon can be partially explained by the organization of general practices (generalization of appointment-only consultation, growth of part-time employment) [18] and also by the insufficient capacity of private practices [19]. There exists a communicating vessel effect between the decrease in non-urgent health structures providing outpatient care and the care provided by EDs, despite the high activity levels of the aforementioned structures [18,20]. This dysfunctional pathway to primary care produces a heavy economic burden for our society [18]. However, the issue is considerably more complex. It is important to know why these patients choose to consult at an ED rather than with their PCP. Scientific literature details several reasons for “inappropriate” consultations at an ED: unavailable PCP [2,12,21,22,23,24], changes in patient behavior regarding primary care access [2,25], patient determination to have additional biological testing or imagery [25,26], lower socio-economic status [27], and unwillingness to pay up front [28,29]. This information could help to determine whether it could be suggested to patients on arrival at the ED to consult their PCP without having been examined by an ED physician.

The aim of this study was to analyze the profiles, motivations, and post-ED consult satisfaction of patients not requiring ED consultation. The aim was also to determine whether it is possible to redirect patients to a PCP before consulting with the emergency physician based on a potential initial triage error. We hypothesized that improved understanding of these patients could lead to appropriate action while stemming the ever-increasing flow of ED patients in France. Characterization of these patients in different environments with different care offerings would provide better understanding of the expectations that lead them to come to the ED. Therefore, based on this characterization and on the potential risk of redirecting patients, we would be able to determine whether an alternative solution is really possible or not without risk to the patient.

## 2. Methods

### 2.1. Study Design

We performed a prospective multicentric study in the ED of Bichat University Hospital in Paris (France) from March 13 until April 16, 2018. This study was also conducted in the ED of Poitiers University Hospital from June 11 until August 5. Each patient consulting an ED in France is ranked by a triage nurse (TN) using the French Emergency Nurses Classification in Hospital scale (CIMU) (Appendix B) [30]. The least severe patients are ranked as CIMU 5, meaning that there is no functional impairment or organic lesion justifying the use of hospital resources. These two sites were chosen because they are in towns with different sizes and locations. However, their organizational structures are comparable insofar as they both use a fast track that is isolated from the rest of the ED for CIMU 5 patients.

### 2.2. Objectives

The primary objective of this study was to determine the profile of CIMU 5 patients consulting the ED, including sociodemographic characteristics, health service access, and reasons for consulting at an ED.

Secondary objectives were to:

- Establish whether CIMU 5 patients could be reoriented by the TN towards their PCP without an ED physician consultation;
- Assess post-ED consultation satisfaction.

### 2.3. Population

In the Bichat University Hospital ED, an average of 250 patients present themselves for consultation each day (90,000 patients per year). Close to one-third of them leave the ED without any further testing or immediate treatment. The hospital is located in the 18th district of Paris, with a population in 2015 of 199,135; it is an urban area suffering from a lack of general practitioners, with 6.5 PCPs per 10,000 inhabitants, with lows of 5.5 and 2.9 PCPs in some of the poorer sectors in 2013 [31]. This is in contrast to the national average of 8.9 PCPs per 10,000 inhabitants in 2016 [32]. In Paris, there are 39 university hospitals, two of which are in the 18th district: Bichat and Bretonneau. In this district, only Bichat has a 24-hour-a-day emergency department. There are also private emergency departments in Paris. Poitiers University Hospital is located in a small city (87,918 inhabitants in 2015). An average of 120 patients are admitted to the ED each day (44,000 per year). There are 126 PCPs in the city, which is a high density at 14.3 PCPs per 10,000 inhabitants. Only the university hospital and a private clinic provide a 24-hour-a-day ED.

Inclusion criteria of patients were:

- Age > 18 years;
- Beneficiary of social security;
- French or English speaking and writing;
- French resident;
- Spontaneously coming to consult at the emergency department; in other words, not having been sent to the ED by the 112 emergency hotline, another physician, or brought to the ED by any first responder (police, fire department, military);
- Categorized as CIMU 5.

Exclusion criteria were:

- Having been asked back by the ED (systematic follow-up, clinical deterioration);
- Unable to write (for example, due to trauma);
- Vulnerable or marginalized members of the population (homeless, refugees, migrants, undocumented migrants, etc.);
- Presenting with a nonmedical issue;
- Having refused to participate in the study;
- Having answered the survey but having left without having been seen by the ED physician;
- Not categorized as CIMU 5 (in case of survey given to the wrong patient);
- Other cases (inaccurate history).

### 2.4. Questionnaire

A survey (Appendix A) was built to gather the required data. Its structure is based on the literature. These findings were validated by a committee of experts using the Delphi method, wherein questions were added, removed, or modified until a consensus of at least 65% agreement was reached [33]. After discussion with the experts during the Delphi process, we voluntarily focused only on patients who had the possibility of consulting elsewhere than in an ED. Presently, France is facing major immigration-related social challenges, but the solution as it pertains to healthcare cannot be found only at the ED level, and requires social and political considerations. Barriers to access to health services are thought to include education, cultural differences, language difficulties, lack of complementary voluntary health insurance, and legal issues [6].

### 2.5. Data Collection

The TN handed out a survey (in French or English) to the included CIMU 5 patients so they could answer it while waiting for their medical consultation. The completed survey was given back to the fast track physician at the end of the visit and the patients were asked about their satisfaction level. Additional clinical data were extracted from the digital patient record using M-UrQual^®^ software (Maincare Solutions, Cestas, France) in the Bichat Hospital ED and ResUrgences^®^ software (Berger Levrault, Boulogne-Billancourt, France) in the Poitiers Hospital ED, including the medical ranking of clinical severity. The patients were contacted by phone 15 days after their ED visit, between 18:00 and 20:00, to collect information about their status once having left the ED and their satisfaction level. We called a given patient 3 times on separate days before considering the person as “non-respondent”.

### 2.6. Flow Chart

**Figure 1:**
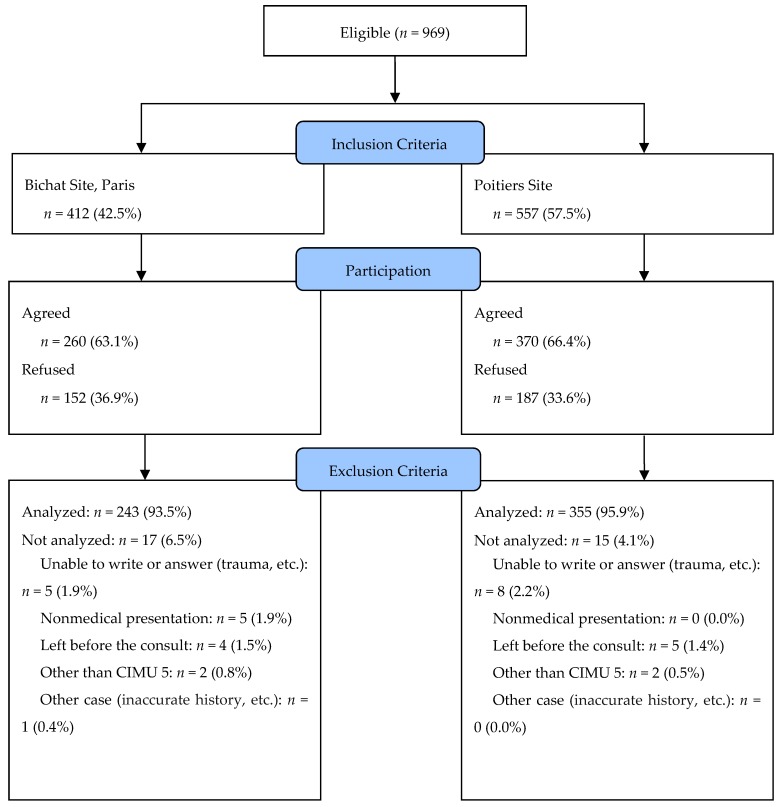
CONSORT (Consolidated Standards of Reporting Trials) 2010 Flow Chart.

At the Bichat University Hospital, 412 patients met the inclusion criteria. They represented 5.2% of the overall activity at the ED. Overall, 260 agreed to answer. Of these, 17 were excluded for various reasons. In total, 243 surveys were gathered, representing a 59.0% answer rate (Figure 1). At the Poitiers University Hospital, 557 patients were included in the study. They represented 8.7% of the total ED activity. Overall, 370 agreed to answer the survey. Of these, 15 were excluded for various reasons. In total, 335 surveys were gathered, representing a 63.7% response rate. Overall, 598 surveys were studied (Figure 1).

### 2.7. Outcomes and Study Variables

The clinical severity of CIMU 5 patients was compared with the French emergency department medical clinical classification (*classification clinique médicale des urgences*, CCMU) [34]. It is used to evaluate the severity of the patient at the end of the clinical examination (Appendix C). We assumed that CIMU 5 patients would be classified less than CCMU 3 (presentation likely to deteriorate without life-threatening prognosis). Patient satisfaction after having left the ED was studied through a Likert 5-level scale (very unsatisfied, unsatisfied, no opinion, satisfied, very satisfied). Variables assessed in the survey (Appendix B) were demographic data (sex, age), place of residence, social security coverage, PCP or not, and professional status as defined by the French National Institute of Statistics and Economic Studies (INSEE), including 8 categories of work and a student category. Number of visits to an ED per year, reason for consulting, date of onset of symptoms, consultation at PCP or other doctor’s office, and motivation to visit an ED were also assessed.

### 2.8. Statistics

Statistical analysis was carried out with the Statview^®^ version 4.5 application (SAS Institute Inc., Cary, NC, USA) and Excel 2016^®^ (Microsoft, Redmond, WA, USA). Sample size calculation was based on the comparison of an assumed observed proportion of 2% CCMU 3 or more among the CIMU 5 patients, with theoretical CIMU 5 patients classified as CCMU 1 or 2. Number of patients to include was estimated at 206 in each center, based on an alpha risk of 0.05, a statistical power of 0.95, and using unilateral tests. Taking into account refusals of participation, exclusions, and unusable data, we assumed that it was necessary to recruit twice the estimated number of patients (i.e., 412). The daily flow of ED visits to Bichat is twice that of Poitiers. We hypothesized that at least one month would be necessary to carry out the study in the Bichat ED and two months in the Poitiers ED. The normality of the distribution for each parameter was explored using the Kolmogorov–Smirnov test. Continuous variables were described using means and standard deviations or medians and first and third quartiles (Q1, Q3). Categorical variables were described using population count and corresponding percentage for each modality. Continuous variables were compared to one another using the Student *t* test or a nonparametric Mann–Whitney U test if necessary. Comparisons between several categorical variables were carried out using the nonparametric Kruskal–Wallis test due to small sample sizes. Categorical variables were compared one-to-one with a Chi-square test. A Spearman test (rho) was used for correlation analysis.

A *p*-value < 0.05 was considered statistically significant.

### 2.9. Ethics

The patients were informed of the study, of it being optional, and of the anonymization of the answers at every step (when the surveys were handed out and during the follow-up phone call). Data collection was done with every survey handed out to each patient. The phone numbers collected in the surveys allowed us to contact the patients afterwards. This procedure was accepted by the *Commission Nationale de l’Informatique et des Libertés* (CNIL) and the Emergency Department committee on ethics, research, and informatics (IRB number U-2017-4.2). The informed consent form is shown in Appendix D. Research was performed in accordance with the Declaration of Helsinki. The local ethics committee of the University Hospital of Poitiers approved this research. Participants consented to participate.

## 3. Results

### 3.1. CIMU 5 Patient Characteristics

A clear majority of the participants were men, who represented 79.4% (*n* = 179) of the patients. The median age was 38 years (interquartile range: 27; 50). In 52.1% of all cases (*n* = 314), they lived near the ED (less than 15 km and less than 20 minutes away by car or mass transit). For 70.7% of patients (*n* = 432), the PCP was near the hospital. In total, 85.5% of the patients (*n* = 511) were covered by social security, while 66.1% of patients (*n* = 395) also had complimentary health coverage. Additionally, 87.1% of patients (*n* = 521) declared a principal PCP. The socio-economic category "employee" was the most widely present at 33.9% (*n*=203), followed by the unemployed, who represented 14.9% (*n* = 89). Characteristics and comparisons between the Bichat and Poitiers sites are presented in Table 1.

### 3.2. Patient Flow Paths

Patient flow paths median time between symptom onset and ED consult was 2 days (interquartile range: 1; 7) at Bichat, and 1 day (interquartile range: 1; 6) at Poitiers. The maximum delay was 730 days for a patient of Bichat suffering from chronic back pain. This patient was excluded from the study because of possibly inaccurate history. At Bichat, only 16.5% (*n* = 40) of patients had consulted a doctor before coming to the ED, whereas 43.9% (*n* = 156) had done so at Poitiers (*p* < 0.0001). At Bichat, 86.0% (*n* = 209) of consultations occurred during hours of the day when private practice alternatives were available. At Poitiers, 81.7% of patients (*n* = 290) consulted during hours of the day when private practice alternatives are available, which was comparable to Bichat (*p* = 0.16). Detailed analyses of the total population and for each hospital for the days and schedules of consultations according to whether or not a PCP was reported are given in Table 2. Patients having a PCP or not consulted similarly during business hours (*p* = 0.57). Additionally, having a PCP did not influence the choice of consulting, either during the week or during the weekends and holidays (*p* = 0.58). There was no difference in behavior between patients who reported or did not report a PCP in both hospitals, either in terms of days or of hours of consultation.

### 3.3. Complaints Justifying the Consultations

All the complaints were sorted out using the French Emergency Medicine Society (SFMU) classification shown in Table 3. There was no difference between the two sites when the categories were compared globally (*p* = 0.32). Osteo-articular pathologies, including rheumatology and traumatology reasons for consultation, were relatively predominant, accounting for 38.3% (*n* = 93) of the total at Bichat and 31.5% (*n* = 112) at Poitiers (*p* = 0.09). Nevertheless, comparison of categories shows that some complaints were voiced at a significantly different rate at the two sites. For instance, at Bichat, gastro-intestinal complaints were more frequent than at Poitiers (*p* < 0.0001), whereas ophthalmological and ear, nose, and throat (ENT) complaints were brought up more frequently at Poitiers (*p* < 0.0001 and *p* = 0.02, respectively). The primary reason for consulting for women was skin issues (29 out of 123 women, or 23.6%), whereas for men it was trauma (62 out of 475 men, or 13.1%). The different reasons for patients to consult at an ED are shown in Table 4. There were no differences between the sites in global comparison of reasons for consultation (*p* = 0.29). The main factor that brought the surveyed patients to consult at an ED was the expectation of getting hospital-based care, including access to further testing or hospitalization. This expectation was shared by 26.8% (*n* = 65) of Bichat patients and by 29.9% (*n* = 106) of Poitiers patients (*p* = 0.68). Patients consulting for traumatology significantly more frequently expected additional testing or imagery than other patients (*p* = 0.003). The proportion of patients mentioning personal convenience (geographic proximity, opening hours) as a justification to consult at an ED differed between the sites. This type of reason concerned 30.9% (*n* = 75) of Bichat patients and 20.2% (*n* = 72) of Poitiers patients (*p* = 0.003). The French health system allows patients not to pay for their care up front after having visited an ED, which is sometimes the case in the private sector. The reasoning of not having to pay on the day of consultation was given by only 5.3% (*n* = 13) of Bichat patients and 2.0% (*n* = 7) of Poitiers patients. The difference between the two sites was significant (*p* = 0.02). In France, the existing system actually encourages patients to be managed in an ED in cases of workplace accidents if they come by ambulance. However, in other cases they have the possibility to consult a PCP. Consequently, “workplace accident” does not systematically mean urgent care for the emergency staff.

### 3.4. Care provided at the ED

At Bichat University Hospital, out of a total of 243 patients, 9.5% (*n* = 23) had laboratory testing, 24.3% (*n* = 59) had imagery, and 5.3% (*n* = 13) had both. Together, 28.4% (*n* = 69) of these patients received further testing. At Poitiers University Hospital, amongst the 355 patients, 11.3% (*n* = 40) had laboratory testing, 28.2% (*n* = 100) had imagery, and 5.9% (*n* = 21) had both. Together, 33.5% (*n* = 119) of these patients underwent further testing. In total, 6.2% (*n* = 15) of Bichat patients received some form of treatment. Among the CIMU 5 patients included in the present study, 2.1% (*n* = 5) were hospitalized after the ED stage. All of them had received laboratory and imagery exploration and some form of treatment. At Bichat, one patient left after completing the survey given by the TN but before seeing an emergency physician and could not be categorized according to the CCMU medical classification, and four did the same at Poitiers. Consequently, 242 and 351 patients were analyzed at Bichat and Poitiers hospitals, respectively. Comparison between the CCMU categories of the CIMU 5 patients at the two sites yielded no difference (*p* = 0.81) (Table 5). In 2.2% of consultations, patients triaged as CIMU 5 by the TN had a medical issue or a functional prognosis that would probably deteriorate during the ED stay (CCMU 3) or that was life-threatening (CCMU 4). All the CCMU 4 and some of the CCMU 3 patients were hospitalized and were not considered as non-urgent.

After their ED visit, 85.6% (*n* = 512) of patients were satisfied or totally satisfied with the care they received. Additionally, 7.2% (*n* = 43) of patients had no opinion, and 7.2% (*n* = 43) of patients were unsatisfied or totally unsatisfied. There was no difference between both sites (*p* = 0.38). Waiting times were deemed satisfactory by 65.7% of the patients. Median waiting time was 71 min (interquartile range: 43; 94) and 73min (interquartile range: 48; 95) at the Bichat and Poitiers University Hospitals, respectively (*p* = 0.24). Level of patient satisfaction was inversely correlated to waiting time (rho = 0.61, *p* < 0.0001).

At 15 days after ED consultation, the patients were contacted by phone. At Bichat, out of the 243 patients, 56.4% (*n* = 137) did not respond, 3.7% (*n* = 9) refused to respond, and 39.9% (*n* = 97) answered our questions. In 90.7% (*n* = 88) of cases, the ED physician had provided the care the patient desired. Out of the 88 patients, 33 reconsulted for the same issue. For 21 of them, it was after an ED physician’s suggestion to consult their PCP for a follow-up consult that they did so. Regarding the patients for whom the ED physician had not provided the desired care, 9 of them consulted at another health facility (*p* < 0.0001). At Poitiers, amongst the 355 patients, 49.0% (*n* = 174) did not answer, 5.1% (*n* = 18) refused to answer, and 45.9% (*n* = 163) agreed to respond. Out of the 163 patients, 41 consulted again for the same reason, as they felt that their ailment had not improved or that the ED physician had not provided the expected care.

## 4. Discussion

### 4.1. Profile and Flow Path of Patients Manageable by General Practice Consultations

The patients who did not need to go to an ED but rather to non-emergency care were young, mostly male, and with adequate social coverage, such as has been reported in other studies [13,26]. As in the study by Piggozo [23], employees and the unemployed were the most widely represented categories. When consulting at an ED, patients used a coherent approach, as described by Gentile et al. 15 years ago [19]. This choice depends on the perception the patient has of an emergency [19]. In our study, more than half consulted less than 24 hours after symptom onset, compared to two-thirds in the study by Gentile in 2010 [26]. Additionally, the comparison between sites in our study suggests that the complaint impelling a person to consult in an ED may be influenced by hospital specificities. If the main complaint was trauma, as has been shown [19,26], ED recourse was similar, whereas frequency of other complaints was site-dependent. At Bichat, for example, there were few ophthalmological or ENT-related issues. This was probably because, being in Paris, other university hospitals contain units dedicated to those specific types of pathologies. At Poitiers, where only one university hospital and one private clinic have an ED, these issues were significantly more present. The motivating factor of personal convenience has also been highlighted. Geographic proximity of the hospital was cited for both sites at a rate of 17.7% (*n*=106) for all studied patients, which is similar to the findings of the 2013 DREES (Research, Studies and Statistics Directorate, Ministry of Social Affaire and Health) French national survey [2]. In the present study, there existed a disparity between the sites, with a higher proportion of such patients at Bichat. This difference could be due to the shortage of general practitioners in the area surrounding Bichat. Accordingly, the number of patients having no declared principal PCP, a French health administration requirement, was significantly higher at Bichat than at Poitiers. Additionally, for those having a principal PCP, the patients at Poitiers were significantly more likely to report that their PCP was located nearby. Other studies have described proximity as being the main factor behind the choice to consult at an ED for more than half of patients [13]. However, this reason does not appear to be the only determining factor. Indeed, although the patients consulting at Bichat were significantly more likely not to have a PCP, there was no difference between Bichat and Poitiers in the number of patients citing unavailability of their PCP as a reason to consult the ED. Finally, the consultations occurred during general practice opening hours, which in the majority of cases corresponded to weekday business hours at both Poitiers and Bichat. Other French studies have shown similar numbers, with 20% to 24% of patients mentioning a lack of PCP availability [2,23,25]. Only one study at a Nimes ED showed that 75% of patients having had no further testing actually encountered difficulties consulting their PCP [22]. Nonetheless, our study showed that even if a patient had ready access to a general practice, they preferred to go directly to an ED, and in most cases without asking for a referral beforehand, as described by Gentile et al. [26]. One recurring reason for consulting at the ED was an expectation of getting further testing and imagery. This could be explained by the predominance of osteo-articular pathologies presenting at the ED. This reasoning has been explored in French studies such as the one conducted in 2016 at a non-teaching hospital, where it was found that 43% of consultations were motivated by a demand for imagery testing [25]. At the national level, trauma is the principal presenting pathology for ED consultations in the general population, with a rate of 36% [2]. The 2010 Health and Social Protection survey reported that in the event of traumatic injury during the week, 9 times out of 10 the patient goes to an ED rather than to a PCP [2]. Traumatology is also the primary reason CIMU 5 patients consult at an ED, ranging from 17% to 63% depending on the study [13,35]. We hypothesize that the patients believe that imagery is systematic and that it is more convenient to have all further testing carried out at an ED, instead of consulting at a PCP, then getting imagery done, and finally consulting at the PCP once again. This factor could explain the mixed results achieved by expensive primary care units (PCUs) in attracting these types of patients [18]. It is for this reason that to alleviate the increasing use of EDs, the French *Cour des Comptes* suggests that non-scheduled, urban-based points of care should be encouraged, possibly by installing testing facilities in PCUs [36]. Furthermore, a recent parliamentary report recommends the facilitating access to the simpler forms of imagery and biological testing in private practices [17]. Gentile et al. demonstrated that these structures could, with the appropriate opening hours, be adapted to fulfill the needs of the population in medical service provision. On the other hand, the often-stated main motivation for consulting being the absence of up-front payment is factually unfounded. Our results suggest that this reason for consulting at an ED is only a minor factor. Nonetheless, at Bichat this reason was stated more often than at the Poitiers CHU in non-severe patients at a rate higher than for the general French population [2]. Poverty, therefore, seems to be a relevant factor. The great majority of patients consulted during business hours regardless of whether or not they had access to a PCP. It was, therefore, not a scheduling issue that was blocking those patients’ access to a PCP. Our results suggest that up to 69.0% of these patients would be interested in another solution than the ED if access to testing facilities were available during the same hours of the day. Most of the CCMU 2 patients, who represented 28.1% of the studied patients, could have consulted their PCP. For these patients, further testing could have been delayed and performed in private practices. Instead, they were done on site because of easy access to testing facilities.

In total, 88.0% of patients consulting an ED of their own volition are categorized as CCMU 1 or 2 according to the 2002 DREES national survey. Expected savings when compared to a non-urgent care structure are, therefore, substantial, but they cannot be achieved without ensuring the geographic proximity of private practice points of care to testing facilities. Indeed, our results showed that 5 patients (2.1%) who were correctly triaged as CIMU 5 by the TN required hospitalization. They showed clinical signs of severity and had normal vital signs. Sending CIMU 5 patients to their PCPs would be a loss of time, and in a few cases of chances of receiving proper care. This is why all CIMU 5 patients must be examined by an emergency physician. A 2003 law enacted in the United States follows the same reasoning—it is forbidden to send home or re-orient a patient solely on triage [1]. Patient satisfaction for ED physicians was 85.6%, which shows that the studied EDs have managed to cope with this demand for nonscheduled care.

### 4.2. What Solutions Could Be Feasible to Optimally Offer Care for These Patients?

All of these data suggest three possible flow paths for the care of CIMU 5 patients. If we consider that these patients must be cared for in an ED environment, then the development of specific fast tracks to welcome them should be encouraged. The corresponding sector must be isolated from the rest of the ED with specific management by a designated physician, so as to avoid disrupting the care of the most severe patients. This type of management could also be done by the PCPs, as long as they have easy access to testing facilities in accordance with patient needs. We speculate that most of the patients who are classified CIMU 5 would receive the same treatment in other university hospitals because they usually do not require further exploration, laboratory testing, or imaging. A small proportion of patients would be referred to hospital after examination and would likely be classified as CCMU 3 or higher. On the other hand, if these patients come directly to the ED, it is impossible to redirect them to another PCP before the medical consultation, since some of the CIMU 5 patients would then classified by the emergency physician as CCMU 3 or higher. In reality, and even if the patient does not require further testing, that patient would not spontaneously come to such a structure if it were lacking the potential for medical testing [26], possibly diminishing the flow of patients to the ED [9]. Xin concluded in a recent study that whenever possible PCPs may be called upon to devote more effort to both communication and quality of care to improve patients’ health outcomes and satisfaction and to reduce non-urgent ED use [37]. Finally, a third path could be laid out by developing hybrid organizations between an ED and one or several general practices. A recent study demonstrated that this solution could be an interesting alternative [38]. Such organizations have been evaluated abroad, such as in Switzerland [39] and Belgium [10]. Their conclusions remain prudent and more studies of these organizations, which seem to attract patients and could help to resolve ED overcrowding, are necessary [9]. This type of management could reduce the number of consultations in EDs by 40% and yield significant improvements of time and resources [40].

Some PCPs judge these types of organizations very favorably because they steer trauma patients directly to an ED [22]. The effectiveness of these solutions cannot depend solely on EDs. Appropriate political means must be set up to resolve the problems raised in the present study. Reforms relating to the emergency system in France should be carried out at the national level. Finally, these organizations cannot impact consultations not requiring an ED in the most vulnerable or marginalized segments of the population. There is an overall increase in ED visits in Europe by people in precarious social situations, such as the homeless, migrants, and asylum seekers. It has been characterized as the result of cultural differences, poor knowledge of local healthcare systems, and language barriers [41,42,43]. In France, health policy specifically targeted at improving the health of migrants is not very developed and has primarily focused on the prevention of infectious diseases such as AIDS and tuberculosis [6]. Access to healthcare other than at an ED should be facilitated at a national and supranational level in Europe in order to address migrants’ health needs.

### 4.3. Limits

This study is not without limitations. Firstly, one bias in our study is linked to the selection of our CIMU 5 patients. We selected only 13.9% of all CIMU 5. There were more eligible participants in Poitiers than in Bichat hospital, i.e., the smaller establishment. The language criterion was the main limitation but arguably selected those most likely to have access to a nearby PCP and to possess knowledge of the healthcare system. Usually patients who cannot speak French or English are migrants or refugees. In the Parisian area of Bichat, there are more migrants and refugees than in Poitiers. In the present study, in order to avoid a cultural bias, they were not included. Consequently, we speculate that this situation may have facilitated inclusions in the hospital at Poitiers compared to those at Bichat. Moreover, the aim of this study was to provide a possible alternative to EDs for these consultations. The problems of refugees and migrants require other political and social considerations, since they currently have little or no alternative to ED consultation. This was not the case for this study population. A future study specific to vulnerable populations should be carried out. Additionally, not all of the patients who met all of the selection criteria were able to be included by the TNs, particularly during periods of very high inflow. We speculated that in overcrowding periods, TNs are less likely to include patients. The ED of Bichat is more exposed to this situation than in Poitiers. Another potential bias is that of the test centers, considering that the urban areas presented different socio-economic makeups. Even though the surveys were anonymous, certain answers may have been influenced by fear of being judged by the caregivers. Finally, some factors associated with inappropriate ED usage were not studied. A recent study in a Japanese hospital found that having two or more prior out-of-hours ED visits in the past 3 years was identified as a factor [14].

## 5. Conclusions

This study has allowed us to demonstrate that users of non-emergency care have a coherent approach when consulting at an ED. Motivating factors for consulting at an ED rather than the PCP seem to be personal convenience, accessibility of emergency facilities, and geographic proximity. The patients are satisfied with care and this satisfaction is inversely correlated to waiting time. They cannot be turned away before having been seen by an ED physician, as at least a small portion of them will require hospitalization. It seems essential that healthcare points of entry with access to testing facilities should put them to work by either employing an internal fast track or by developing “hybrid” health structures by associating an ED with one or several local PCP structures, thereby helping to resolve ED overcrowding.

## Figures and Tables

**Table 1:** Characteristics of patients who did not require an emergency department (ED) level of care.

Characteristics of Patients	Total *n* = 598	Bichat *n* = 243	Poitiers *n* = 355	*p*
**Male patients**, no. (%)	475 (79.4)	179 (73.7)	296 (83.4)	0.003
**Age** (year), median (IQR *)	38 (27–50)	37 (27–50)	38 (26–49)	0.74
**Nearby place of residence** **, no. (%)	314 (52.5)	140 (57.6)	174 (49.0)	0.04
**Basic Social Security coverage**, no. (%)	511 (85.5)	200 (82.3)	311 (87.6)	0.07
**Complementary health coverage**, no. (%)	395 (66.1)	142 (58.4)	253 (81.4)	0.04
**Designated primary care provider**, no. (%)	521 (87.1)	189 (77.8)	332 (93.5)	<0.0001
**Nearby primary care provider**, no. (%)	423 (70.7)	215 (88.5)	208 (58.6)	<0.0001
**Professional category/occupation**, no. (%)				0.27
Farmers	5 (0.8)	1 (0.4)	4 (1.1)	
Managers	50 (8.4)	29 (11.9)	21 (5.9)	
Craftsman/woman	33 (5.5)	10 (4.1)	23 (6.5)	
Laborers	48 (8.0)	21 (8.6)	27 (7.6)	
Middle management	41 (6.9)	7 (2.9)	34 (9.6)	
Employees	66 (11.0)	22 (9.1)	44 (12.4)	
Inactive	89 (14.9)	39 (16.1)	50 (14.1)	
Students	203 (33.9)	86 (35.4)	117 (32.9)	
Retired	63 (10.6)	28 (11.5)	35 (9.9)	

Note: * IQR: interquartile range; ** < 15 km and < 20 min away from the hospital by personal vehicle or by mass transit.

**Table 2:** Day and time of consultations not requiring an ED level of care.

	Total of CIMU 5 * Patients			University Hospital of Bichat			University Hospital of Poitiers		
	PCP **	No PCP	*p*	PCP	No PCP	*p*	PCP	No PCP	*p*
08:00–20:00	433	66	0.57	161	48	0.48	272	18	0.66
20:00–08:00	88	11		28	6		60	5	
Weekdays	363	56	0.58	145	40	0.69	218	16	0.15
Weekends/Holidays	158	21		44	14		114	7	

Note: * CIMU: The French Emergency Nurses Classification in Hospital scale (CIMU 5: no functional impairment or organic lesion justifying the use of hospital resources); ** PCP: primary care provider.

**Table 3:** Consultation reasons given by CIMU 5 * patients as categorized by SFMU **.

Consultation Reasons	Total *n* = 598	Bichat *n* = 243	Poitiers *n* = 355	*p*
**Cardio-vascular**, no. (%)	26 (4.3)	13 (5.3)	13 (3.7)	0.32
**Dermatological**, no. (%)	74 (12.4)	23 (9.5)	51 (14.4)	0.07
**Gastroenterological**, no. (%)	43 (7.2)	30 (12.4)	13 (3.7)	<0.0001
**General and other**, no. (%)	58 (9.7)	30 (12.4)	28 (7.9)	0.07
**Urogenital**, no. (%)	24 (4.0)	13 (5.3)	11 (3.1)	0.17
**Neurological**, no. (%)	28 (4.7)	10 (4.1)	18 (5.1)	0.59
**Ophthalmological**, no. (%)	57 (9.5)	4 (1.6)	53 (14.9)	<0.0001
**ENT**, no. (%)	67 (11.2)	18 (7.4)	49 (13.8)	0.02
**Psychiatry**, no. (%)	7 (1.2)	4 (1.6)	3 (0.8)	0.45
**Respiratory**, no. (%)	9 (1.5)	5 (2.1)	4 (1.1)	1
**Rheumatology**, no. (%)	109 (18.2)	49 (20.2)	60 (16.9)	0.31
**Traumatology**, no. (%)	96 (16.1)	44 (18.1)	52 (14.6)	0.26

Note: * CMIU: The French Emergency Nurses Classification in Hospital scale (CIMU 5: no functional impairment or organic lesion justifying the use of hospital resources); ** SFMU: French Society of Emergency Medicine.

**Table 4:** Motivation of patients without functional impairment or organic lesion justifying the use of hospital resources for consulting an ED.

Motivation	Total *n* = 598	Bichat *n* = 243	Poitiers *n* = 355	*p*
**Workplace accident**, no. (%)	17 (2.8)	6 (2.5)	11 (3.1)	0.65
**Suggested by peers**, no. (%)	3 (0.5)	1 (0.4)	2 (0.6)	1.0
**Suggested by a professional** *, no. (%)	58 (9.7)	17 (7.0)	41 (11.6)	0.06
**Second opinion**, no. (%)	21 (3.6)	7 (2.9)	14 (3.9)	0.49
**Intense pain**, no. (%)	27 (4.5)	9 (3.7)	18 (5.1)	0.43
**Additional testing**, no. (%)	157 (26.3)	59 (24.3)	98 (27.6)	0.36
**Appointmenthours**, no. (%)	8 (1.3)	4 (1.6)	4 (1.1)	0.59
**After business hours** **, no. (%)	31 (5.2)	8 (3.3)	23 (6.5)	0.11
**Hospitalization**, no. (%)	14 (2.3)	6 (2.5)	8 (2.3)	0.86
**Unavailable PCP** ***, no. (%)	115 (19.2)	42 (17.3)	73 (20.6)	0.32
**Lack of upfront payment**, no. (%)	22 (3.7)	13 (5.3)	9 (2.5)	0.07
**Geographic proximity** ****, no. (%)	106 (17.7)	63 (26.0)	43 (12.1)	<0.0001
**Already taken care of in this hospital**, no. (%)	19 (3.2)	8 (3.2)	11 (3.0)	0.89

Note: * Primary care provider, other physician, paramedical professional; ** 20:00–08:00; *** primary care provider; **** < 15 km and < 20 min away from the hospital by personal vehicle or by mass transit.

**Table 5:** Medical classification of the degree of severity (CCMU *) after medical examination of patients categorized CIMU 5 ** by the triage nurse.

Clinical Severity	Total *n* = 593	Bichat *n* = 242	Poitiers *n* = 351	*p*
**CCMU 1**, no. (%)	399 (67.3)	167 (69.1)	232 (66.1)	0.81
**CCMU 2**, no. (%)	178 (30.0)	68 (28.1)	110 (31.3)	
**CCMU 3**, no. (%)	10 (1.7)	4 (1.7)	6 (1.7)	
**CCMU 4**, no. (%)	3 (0.5)	1 (0.4)	2 (0.6)	
**CCMU 5**, no. (%)	0 (0.0)	0 (0.0)	0 (0.0)	
**CCMU P**, no. (%)	3 (0.5)	2 (0.8)	1 (0.3)	

Note: * CCMU: (French) clinical classification of emergency department patients (*classification clinique médicale des urgences*); ** CMIU: The French Emergency Nurses Classification in Hospital scale (CIMU 5: no functional impairment or organic lesion justifying the use of hospital resources); CCMU 1: stable situation, abstention from complementary diagnostic or therapeutic acts; CCMU 2: stable presentation, requiring a complementary diagnostic or therapeutic act; CCMU 3: presentation likely to deteriorate without life-threatening prognosis; CCMU 4: prognosis committed, no immediate resuscitation maneuver; CCMU 5: prognosis committed, perform immediate resuscitation maneuver. CCMU P: Patient with psychological or psychiatric problems dominant in the absence of any unstable somatic pathology.

## Data Availability

All data analyzed during this study are included in the manuscript and supplemental files. Materials described in the manuscript, including all relevant raw data, are freely available to any scientist wishing to use them for noncommercial purposes, without breaching participant confidentiality. For more details, please contact the corresponding author.

## Abbreviations

CCMU	French clinical emergency department classification (Classification Clinique des Malades aux Urgences)
CIMU	French Emergency Nurses Classification in Hospital scale, CIMU (Classification Infirmière des Malades aux Urgences)
ED	emergency department
ENT	ear, nose, and throat
PCP	primary care provider
SFMU	French Emergency Medicine Society
TN	triage nurse

## Appendix A. The French Emergency Nurses Classification in Hospital scale, Classification Infirmière des Malades aux Urgences (CIMU)

**Table:**

Triage	Description	Action
1	Immediately life-threatening.	Actions focused on support of one or more vital functions. Immediate medical and paramedical intervention.
2	Marked impairment of a vital organ or imminently life-threatening or functionally disabling traumatic lesion.	Actions focused on treatment of the vital function or traumatic lesion. Immediate paramedical and medical intervention within 20 min.
3	Functional impairment or organic lesions likely to deteriorate within 24 h or complex medical situation justifying the use of several hospital resources.	Multiple actions focused on diagnostic evaluation and prognostic evaluation in addition to treatment. Medical intervention within 60 min ± followed by paramedical intervention.
4	Stable, noncomplex functional impairment or organic lesions, but justifying the urgent use of at least one hospital resource.	Consult with limited diagnostic or therapeutic procedures. Medical intervention within 120 min ± followed by paramedical intervention.
5	No functional impairment or organic lesion justifying the use of hospital resources.	Consult with no diagnostic or therapeutic procedure. Medical intervention within 240 min.
*	Intense symptom or abnormal vital parameter justifying rapid corrective action.	Specific action within 20 min. The star can complete a triage 3 or 4.

From: Taboulet et al. [30].

## Appendix B. Survey Distributed to the Included CIMU 5 Patients

Family name: _____________________________________		
First name: _____________________________________		
Sex: ▯ Female ▯ Male		
Birthday:	___ / ___ / ______	
Phone:	__ __ __ __ __	
City/Town of residence:	____________________________	

**1 What is your social security coverage?**

▯ Social security insurance
▯ Complementary health coverage
▯ Special social security (CMU, AME)
▯ None

**2 Do you have a General Practitioner/Family Physician?**

▯ Yes, in which city is your GP? ________________________
▯ No

**3 What is your profession?**

▯ Farmer, agricultural worker
▯ Craftsman, merchant, manager
▯ Executive, intellectual profession
▯ Intermediate professions
▯ Employee
▯ Unskilled laborer
▯ Retired
▯ No professional activity
▯ Student

**4 How many times a year do you visit an emergency department?**

________________________________________

**5 Reason for consultation at the emergency department**

________________________________________

**6 Date of onset of the troubles?**

________________________________________

**7 Have you ever previously consulted for the same reason?** ▯ Yes ▯ No

If yes, how long ago? ______________________

If so, at your doctor’s office?

▯ Yes
▯ No, where did you consult? _______ ________________________________

Have you tried to treat yourself? ▯ Yes ▯ No

**8 Why did you choose the emergency department?**

▯ Proximity to home or work
▯ Schedule compatibility with my activity
▯ Attending GP unavailable
▯ The appointment proposed by my doctor was not convenient for me
▯ I have moved and I have not yet found a doctor
▯ I don’t have a GP
▯ I am on business travel, so I do not have access to my GP
▯ After advice from my GP or another health professional (another doctor, physiotherapist, nurse, etc.)
▯ I wanted another medical opinion, in addition to that of my GP
▯ I would like to have direct advice from a specialist
▯ I thought I would need additional tests (blood test, imagery, etc.)
▯ I thought I would have to be hospitalized
▯ I’m being followed in the hospital
▯ I chose to consult at the emergency department because it is well known that it is the quickest
▯ I cannot pay any upfront fees
▯ Other:

## Appendix C. French Clinical Emergency Department Classification, Classification Clinique des Malades aux Urgences (CCMU)

**Table:**

Classification	Description
CCMU 1	Stable situation, abstention from complementary diagnostic or therapeutic act
CCMU 2	Stable situation, perform a complementary diagnostic or therapeutic act
CCMU 3	Situation likely to deteriorate without being life-threatening
CCMU 4	Prognosis committed, no immediate resuscitation maneuver
CCMU 5	Prognosis committed, perform immediate resuscitation maneuver
CCMU P	Patient with psychological or psychiatric problems dominant in the absence of any unstable somatic pathology
CCMU D	Patient dies at the entrance to the emergency

From: Afilal [34].

## Appendix D. Informed Consent

Dear Sir or Madam,

You are going to see an emergency physician in the fast track unit of the emergency department. A study aimed at evaluating the motivations of patients without life-threatening conditions who use an emergency department is being conducted in the emergency departments of the Bichat-Claude-Bernard and Poitiers University Hospitals. We have also added a patient satisfaction survey. This study is focused on the improvement of the quality of care in the fast track unit of our emergency department. If you agree to take part in it, you will be required to answer this survey during the waiting time after having seen the triage nurse and before seeing the emergency physician.

In this survey, the following data are collected:

- Sociodemographic categories
- Medical administrative information
- Motivation to consult in the emergency department
- Patient satisfaction

The processing of all these data will be strictly confidential and anonymous.

The Emergency Department of Bichat has specific software intended for patient management. The registered information is reserved for the emergency department and can only be communicated to the following addresses:

- Dr Aiham Daniel GHAZALI, main investigator, Emergency Department of Bichat Hospital
- Dr Arnaud CHAUDET, Emergency Department of Poitiers Hospital

You will be contacted by phone 15 days after your ED visit, between 18:00 and 20:00, to collect information about your status once having left the ED and your satisfaction level.

Dr GHAZALI is at your disposal to provide any information which you might consider useful.

According to articles 39 and following articles of law *n* 78-17 of January 6, 1978, modified in 2004, relative to computing, to files, and to personal liberties, any person can obtain the communication, and where necessary, rectification or deletion of their corresponding information, by contacting Dr Aiham Daniel Ghazali (Bichat Hospital, 46 rue Henri Huchard, 75018 Paris).

If you agree to participate in the present survey, a copy of this document will be provided to you and a second signed copy will be given to the emergency physician with the questionnaire.
